# *Wolbachia* Screening in *Aedes aegypti* and *Culex pipiens* Mosquitoes from Madeira Island, Portugal

**DOI:** 10.3390/insects16040418

**Published:** 2025-04-15

**Authors:** Rita Fernandes, Tiago Melo, Líbia Zé-Zé, Inês C. Freitas, Manuel Silva, Eva Dias, Nuno C. Santos, Bruna R. Gouveia, Gonçalo Seixas, Hugo Costa Osório

**Affiliations:** 1 CEVDI-INSA, Centre for Vectors and Infectious Diseases Research, National Institute of Health Doutor Ricardo Jorge, Avenida da Liberdade n.-5, 2965-575 Águas de Moura, Portugal; tiago.melo@insa.min-saude.pt (T.M.); libia.zeze@insa.min-saude.pt (L.Z.-Z.); ines.freitas@insa.min-saude.pt (I.C.F.); manuel.silva@insa.min-saude.pt (M.S.); 2 Center for the Study of Animal Science (CECA)/Institute for Agricultural and Agroalimentary Science and Technology (ICETA), University of Porto, 4051-401 Porto, Portugal; 3 GIMM—Gulbenkian Institute for Molecular Medicine, Av. Prof. Egas Moniz, 1649-028 Lisbon, Portugal; eva.dias@gimm.pt (E.D.); nuno.santos@gimm.pt (N.C.S.); goncalo.seixas@gimm.pt (G.S.); 4 Faculdade de Medicina, Universidade de Lisboa, Av. Prof. Egas Moniz, 1649-028 Lisbon, Portugal; 5 Direção Regional da Saúde, Rua 31 de Janeiro, nº. 54 e 55, 9054-511 Funchal, Portugal; bruna.gouveia@madeira.gov.pt; 6 Interactive Technologies Institute—LARSyS, Polo Científico e Tecnológico da Madeira, Caminho da Penteada, Piso-2, 9020-105 Funchal, Portugal; 7 Environment and Infectious Diseases Research Group, Environmental Health Institute (ISAMB), Av. Prof. Egas Moniz, Ed. Egas Moniz, Piso 0, Ala C, 1649-028 Lisboa, Portugal

**Keywords:** biological control, endosymbiotic bacteria, vector control, *Wolbachia*

## Abstract

Mosquito-borne diseases such as dengue and West Nile virus pose serious public health risks. On Madeira Island, the presence of the mosquito species *Aedes aegypti* (Linnaeus, 1762) and *Culex pipiens* (Linnaeus, 1758) raises concerns about local transmission. In this study, we tested 100 *Ae. aegypti* and 40 *Cx. pipiens* mosquitoes collected exclusively in the municipality of Funchal, Madeira Island, to assess the presence and diversity of *Wolbachia*, a naturally occurring bacterium known to reduce mosquitos’ ability to transmit viruses. Molecular identification confirmed that all *Cx. pipiens* specimens belonged to the *molestus* biotype, with three individuals identified as hybrids between *molestus* and *pipiens* biotypes. This is the first evidence of such hybrids in Madeira. *Wolbachia* was not detected in any of the *Ae. aegypti* samples. In contrast, all *Cx. pipiens* mosquitoes were positive, showing a 100% prevalence. Genetic characterization placed these infections within the *w*Pip clade, supergroup B, sequence type 9. These findings provide key baseline data to inform future mosquito control strategies on the island. As *Ae. aegypti* showed no natural *Wolbachia* infection, introducing *Wolbachia*-infected mosquitoes may be necessary to implement such biocontrol approaches in Madeira.

## Simple Summary

Mosquitoes can spread serious diseases like dengue and West Nile virus. On Madeira Island, two mosquito species—*Aedes aegypti* and *Culex pipiens*—are present and may pose a risk to public health. Scientists are exploring new ways to control these mosquitoes using a natural bacterium called *Wolbachia*, which can reduce a mosquito’s ability to transmit viruses and even lower mosquito populations. However, for these methods to work, it is important to know first if the mosquitoes in the area already carry this bacterium. In this study, we tested *Ae. aegypti* and *Cx. pipiens* from Madeira for *Wolbachia*. *Wolbachia* was absent in all 100 *Ae. aegypti* tested but present in all 40 *Cx. pipiens*. We also found that the *Wolbachia* in *Cx. pipiens* belonged to a group commonly seen in other parts of the world. These results are important because they help us understand which mosquito control strategies might work in Madeira. Specifically, if scientists want to use *Wolbachia* to control *Ae. aegypti* on the island, they would need to introduce it artificially. This information can help improve public health efforts and reduce the risk of mosquito-borne diseases in the region.

## 1. Introduction

*Ae. aegypti* (Linnaeus, 1762) and *Cx. pipiens* (Linnaeus, 1758) are mosquito species of medical and veterinary importance [1,2]. *Ae. aegypti* is recognized as the main vector of several arboviruses—including dengue, Zika, and chikungunya viruses [1]—while *Cx. pipiens* is a well-established transmitter of West Nile virus (WNV) [2]. These diseases pose significant public health challenges, with an estimated four billion people at risk of arbovirus infections worldwide [3]. Over the past five decades, the rapid growth of populations, expanding urban areas, increased travel, and the rising resistance to both larvicidal and adulticidal insecticides have collectively driven the spread of mosquito-borne diseases (MBD) worldwide [4,5,6,7]. Recent environmental shifts—such as rising temperatures, urban expansion, and enhanced global mobility—have contributed to the broader distribution and activity of mosquito populations. The number of human cases derived from MBD has risen significantly in Europe, particularly in Central and Mediterranean regions [8]. The spread of *Ae. aegypti* to Madeira Island, Portugal, in 2005 triggered the first dengue outbreak in 2012, underscoring the island’s vulnerability to future dengue epidemics [9]. Similarly, the presence of *Cx. pipiens* on the island raises concerns about the potential for local WNV transmission, with similar patterns observed elsewhere in Europe [10].

Over the past ten years, *Wolbachia* has emerged as a promising strategy for controlling mosquito-borne diseases [11]. *Wolbachia* is an intracellular endosymbiotic bacterium that lives within arthropods and nematodes and often interferes with host reproduction and/or blocks the transmission of arboviruses such as dengue, Zika, and chikungunya viruses [12]. The most common form of this interference is cytoplasmic incompatibility (CI), in which mating between *Wolbachia*-infected males and uninfected females results in reduced embryo viability. Additionally, in some hosts, *Wolbachia* can induce processes like parthenogenesis (the development of offspring from unfertilized eggs), feminization (transforming genetically male individuals into females), or even the elimination of male embryos [13]. Since *Wolbachia* is maternally transmitted, these reproductive modifications ensure that a greater proportion of females in the population become carriers of the infection. Field trials have explored two main approaches: one aimed at suppressing mosquito populations by releasing infected males and another aimed at replacing the target population with infected individuals of both sexes [8]. To curb dengue transmission in communities, programs releasing *Wolbachia*-infected mosquitoes are currently underway across multiple countries: the United States [14], Brazil [15], Italy [16], Australia [17], Vietnam [18], Indonesia [19], Singapore [20], China [21], and Malaysia [22]. Studies employing this approach have demonstrated significant reductions in dengue incidence, with suppression rates of 40% in Kuala Lumpur (Malasia) [22], over 70% in Yogyakarta (Indonesia) [19], and up to 96% in northern Queensland (Australia) [17]. These findings highlight the potential of *Wolbachia*-based strategies as a sustainable and environmentally friendly alternative for global mosquito-borne disease control.

Despite *Wolbachia*’s proven efficacy in reducing pathogen transmission, there are critical gaps in our understanding of its prevalence and genetic diversity in mosquito populations on Madeira Island. Previous studies have focused on the genetic structure, insecticide resistance, and vector competence of *Ae. aegypti* in Madeira [9,23,24], but no research to date has examined *Wolbachia* prevalence in this population. Given that *Ae. aegypti* populations in Madeira have demonstrated resistance to pyrethroids and organophosphates [24], alternative vector control strategies—such as *Wolbachia*-based approaches—may be necessary to mitigate future arbovirus outbreaks.

This study aims to fill this knowledge gap by screening local populations of *Ae. aegypti* and *Cx. pipiens* for *Wolbachia* infection using molecular techniques, including amplification of the *w*sp gene and Multilocus Sequence Typing (MLST), to genotype and differentiate *Wolbachia* strains. By determining infection rates and strain diversity, this research will provide essential insights into *Wolbachia* dynamics in Madeira’s mosquito populations and inform future vector control programs on the island.

## 2. Materials and Methods

### 2.1. Mosquito Collection

**Figure 1:**
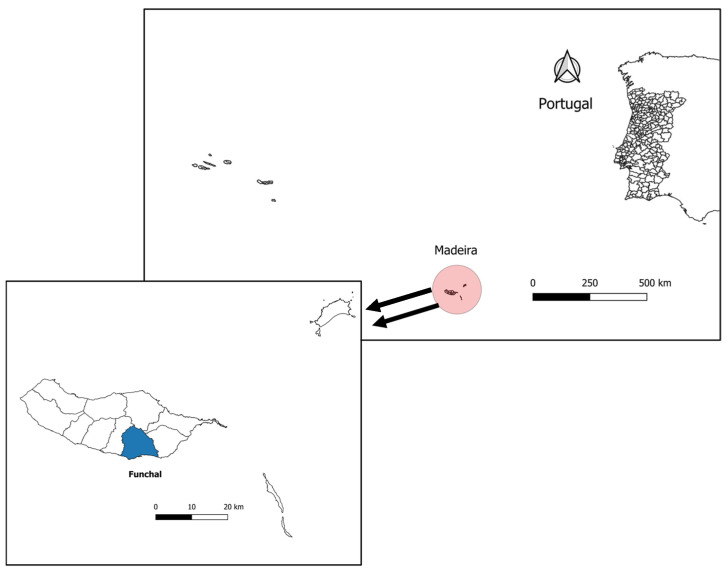
Map showing the geographic location of Madeira Island, Portugal. The highlighted area represents Funchal, where mosquito traps were placed within the municipal limits.

Adult *Cx. pipiens* mosquitoes were collected in August 2023 and August 2024 using BG-Sentinel traps (Biogents, Regensburg, Germany). These traps were strategically placed across various urban and suburban locations within the municipal limits of Funchal, including residential areas and public gardens, where mosquito activity was known to be high. Figure 1 illustrates the geographic location of Madeira Island, with Funchal highlighted as the mosquito collection site. From each year’s collection, 10 males and 10 females were randomly selected, resulting in a total of 40 specimens. Captured mosquitoes were placed in 0.5 mL Eppendorf tubes containing RNAlater (Thermo Fisher Scientific, Waltham, MA, USA) and were stored at room temperature for transport.

Regarding *Ae. aegypti* mosquitoes, eggs were collected in 2023 from multiple oviposition traps—ovitraps–distributed across various sites within the municipality of Funchal. The collected eggs, representing offspring from numerous females, were transported to the laboratory and submerged in water under controlled insectary conditions (28 °C temperature and 80% humidity) until hatching. Emerging adults were maintained under identical standard conditions prior to analysis, ensuring a heterogeneous and representative sample for *Wolbachia* screening. The emerging adults were kept under the same standard insectary settings. To ensure consistency, we analyzed 50 males and 50 females, all tested within 2–4 days post emergence to minimize any age-related physiological differences.

Species identification was conducted under a stereo microscope (SZX7, Stereo Microscope, Olympus LS, Hachioji, Tokyo.) using the Ribeiro and Ramos (1999) identification key, which allowed for the precise differentiation of both *Cx. pipiens* and *Ae. aegypti* from other mosquito species present in the region [25].

Whole mosquitoes were used for individual genomic DNA extractions with the NzyTech Tissue gDNA Isolation Kit (NzyTech, Lisbon, Portugal). The extraction process followed the manufacturer’s protocol. DNA samples were stored at −20 °C until further analysis.

For species identification within the *Cx. pipiens* complex, PCR amplification of the acetylcholinesterase-2 (*ace-2*) gene was performed using the primers ACEpip-F (5′-GGAAACAACGACGTATGTACT-3′), ACEquin-F (5′-CCTTCTTGAATGGCTGTGGCA-3′), and B1246s-R (5′-TGGAGCCTCCTCTTCACGG-3′), following the protocol described by Smith and Fonseca [1]. The PCR reaction mix consisted of 10 µL of NZY Master Mix (NZYTech, Lisbon, Portugal), 0.8 µM of ACEquin-F and B1246s-R primers, 0.4 µM of ACEpip-F primer, 1 µL of DNA template, and water to a final volume of 20 µL. The amplification protocol included one cycle at 94 °C for 5 min, 35 cycles at 94 °C for 30 s, 55 °C for 30 s, 72 °C for 1 min, and a cycle at 72 °C for 5 min. In this approach, *Cx. pipiens* produces a 610 bp fragment, while *Culex quinquefasciatus* generates a 274 bp fragment, allowing for clear species differentiation [26].

To further distinguish between *Cx. pipiens* biotypes and their hybrids, the CQ11 microsatellite locus was amplified using the forward primer CQ11F2 (5′-GATCCTAGCAAGCGAGAAC-3′) and the reverse primers pipCQ11R (5′-CATGTTGAGCTTCGGTGAA-3′) and molCQ11R (5′-CCCTCCAGTAAGGTATCAAC-3′), following the protocol outlined by Bahnck and Fonseca [27]. The polymerase chain reaction (PCR) mix contained 10 µL of NZY Master Mix (NZYTech, Lisbon, Portugal), 0.5 µM of each primer, 1 µL of DNA template, and water to a final volume of 20 µL. PCR conditions were as follows: one cycle at 94 °C for 3 min, 40 cycles at 94 °C for 30 s, 54 °C for 30 s, and 72 °C for 40 s, and one cycle at 72 °C for 5 min. The *Cx. pipiens* has two distinct biotypes: biotype *pipiens* and biotype *molestus*. The different biotypes are represented by the amplification of a 200 bp band for *Cx. pipiens* biotype *pipiens* and a 250 bp band for *Cx. pipiens* biotype *molestus*. For hybrids of *Cx. pipiens* biotype *pipiens* and *Cx. pipiens* biotype *molestus*, the two bands specific for each biotype are simultaneously amplified (200 bp and 250 bp).

### 2.2. Wolbachia Detection

*Wolbachia* detection in mosquito samples was performed using two separate PCR protocols. The primary screening targeted a 610 bp region of the *wsp* gene using primers 81F (5′-TGGTCCAATAAGTGATGAAGAAAC-3′) and 691R (5′-AAAAATTAAACGCTACTCCA-3′), following the method described by Zhou et al. [28]. Reactions were prepared in a total volume of 10 µL, consisting of 5 µL of NZY Master Mix (NZYTech, Lisbon, Portugal), 0.25 µL of each primer (10 µM), 1 µL of DNA template, and 3.5 µL of PCR-grade water. The cycling conditions were as follows: 94 °C for 2 min; 37 cycles of 94 °C for 30 s, 54 °C for 45 s, and 72 °C for 90 s; and final extension at 72 °C for 10 min.

To confirm the absence of *Wolbachia* in samples that tested negative for *wsp*, an additional PCR targeting the 16S rDNA gene was performed using primers WspecF (5′-AGCTTCGAGTGAAACCAATTC-3′) and WspecR (5′-GAAGATAATGACGGTACTCAC-3′), following the method described by Sawasdichai et al. [29]. For this reaction, the master mix composition in a 9 µL reaction was as follows: 5 µL of NZYTaq II (2×), 0.4 µL of each primer (10 µM), 1 µL of DNA template, and 3.2 µL of PCR-grade water. The cycling conditions were as follows: initial denaturation at 95 °C for 3 min; 35 cycles of 94 °C for 30 s, 55 °C for 30 s, and 72 °C for 45 s; final extension at 72 °C for 10 min; and hold at 12 °C.

Amplified products from both protocols were resolved on 2% agarose gels stained with GreenSafe (NZYTech, Lisbon, Portugal) and visualized under UV illumination.

A total of 100 *Ae. aegypti* and 40 *Cx. pipiens* mosquitoes were screened for *Wolbachia*. Samples were classified as positive or negative based on the presence or absence of the expected amplicons from both *wsp* and 16S rDNA-based reactions.

### 2.3. Multilocus Sequence Typing

MLST was performed on *wsp*-positive mosquitoes, targeting five conserved housekeeping genes (*gatB*, *coxA*, *hcpA*, *ftsZ*, and *fbpA*) to characterize *Wolbachia* strains present in the samples [30]. Additionally, the *wsp* hypervariable region (*wsp*HVR) was amplified to provide further strain differentiation. The primer pairs used for each locus, along with the corresponding amplicon sizes, are provided in Table A1.

PCR reactions were conducted using a Veriti™ Thermal Cycler (Applied Biosystems, Foster City, CA, USA), following the same reaction mix conditions as for the *wsp* amplification. The thermal cycling conditions were optimized for each gene. For *gatB*, *hcpA*, *ftsZ*, and *coxA*, the cycle consisted of initial denaturation at 94 °C for 30 s, followed by 37 cycles of 54 °C for 45 s and 72 °C for 90 s. For *fbpA*, the annealing temperature was set at 55 °C for 45 s, with all reactions including a final extension step at 72 °C for 10 min.

All PCR products were analyzed via 2% agarose gel electrophoresis, stained with GreenSafe (NZYTech, Lisbon, Portugal), and visualized under UV light. Successfully amplified DNA fragments were purified using the ExoProStar™ 1-Step PCR Purification Kit (Cytiva, Marlborough, MA, USA) before sequencing.

A total of 20 *Cx. pipiens* specimens (5 males and 5 females from 2023, and 5 males and 5 females from 2024), including all three hybrid individuals identified (two from 2023 and one from 2024), were selected for sequencing. Bidirectional sequencing was performed for two representative samples from each year to improve sequence accuracy and obtain consensus sequences. Sequencing was carried out using an ABI 3130xl Genetic Analyzer (Applied Biosystems, Foster City, CA, USA). Raw sequencing reads were quality-checked, and base-calling errors were corrected using BioEdit (version 7.2.5) [31]. Individual loci (*gatB*, *coxA*, *fbpA*, *ftsZ, hcpA*, and *wsp*HVR) were aligned and concatenated to generate a complete MLST profile. Consensus sequences were submitted to GenBank, and both individual loci and concatenated sequences were queried in the *Wolbachia* MLST database (https://pubmlst.org/wolbachia/, accessed on 10 February 2024) to determine sequence type (ST) assignments.

### 2.4. Phylogenetic Analysis

Homology searches were performed using the BLASTN algorithm [32], and all partial sequences were aligned with via ClustalW in BioEdit (version 7.2.5) [31]. For the integration of *Wolbachia* circulating in the study area into the global genetic diversity, the obtained nucleotide concatenated consensus sequences of MLST loci (*coxA*, *gatB*, *ftsZ*, *fbpA*, *hcpA*) and the *wsp* hypervariable region were aligned against multiple sequences available at PubMLST (https://pubmlst.org/wolbachia/, accessed on 10 February 2024) and manually inspected using BioEdit (version 7.2.5). The obtained nucleotide alignments were further used to build maximum-likelihood phylogenetic trees, applying the obtained best-fit model for each alignment using MEGA 11 [33].

## 3. Results

### 3.1. Mosquito Morphological Identification

In total, 140 mosquitoes from two genera, *Aedes* and *Culex*, were sampled for testing. Among them, 100 samples were identified as *Ae. aegypti* and were evenly distributed between 50 females and 50 males.

For *Cx. pipiens,* 40 field-collected mosquitoes were morphologically identified and selected for DNA testing. The ace-2 fragment-size analysis provided no evidence of *Cx. quinquefasciatus* in all 40 samples; instead, all of them had fragments of 610 bp, corresponding to the size of *Cx. pipiens* molecular amplification ID [26,34]. Based on the CQ11 fragment-size analysis, 37 specimens were assigned to *Cx. pipiens* biotype *molestus* as they all exhibited the 250 bp specific fragment. Additionally, three samples were identified as hybrids [27].

### 3.2. Wolbachia Screening Through Amplification of the Wsp Gene

A total of 140 mosquitoes were screened for *Wolbachia* presence through PCR amplification targeting the *wsp* gene. None of the 100 *Ae. aegypti* mosquitoes tested positive for *Wolbachia* presence. In contrast, 100% of *Cx. pipiens* samples from both 2023 and 2024 collections tested positive for *Wolbachia* infection. To confirm these findings, *Ae. aegypti* samples were also screened using 16S rRNA primers (WspecF and WspecR), which similarly yielded no amplification.

### 3.3. Multilocus Sequence Typing (MLST)

All five MLST loci (*gatB*, *coxA*, *ftsZ*, *fbpA*, and *hcpA*) and the *wsp*HVR were successfully sequenced for all samples.

**Figure 2:**
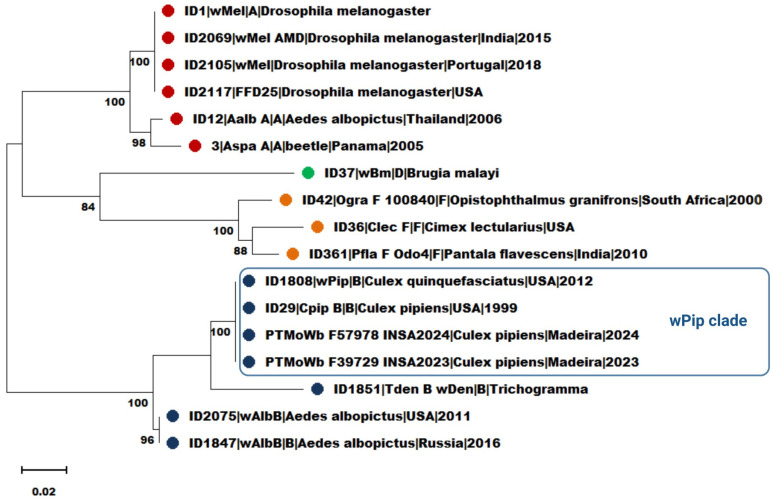
Phylogenetic tree inferred using the maximum likelihood method and Tamura–Nei model (with discrete Gamma distribution and some evolutionary invariable sites; TN93+G+I) from 17 (2 novel) concatenated sequences of MLST loci and the *wsp* hypervariable region obtained from mosquitoes circulating in Funchal (Table A2) and 15 sequences available in PubMLST. Bootstrap values (1000 replicates) are shown below the branches. The analysis involved 2621 positions in the final dataset. *Wolbachia* sequences were identified based on PubMLST ID numbers, insect species, supergroup, country region, country, and year of collection (if available). *Wolbachia* supergroups are presented with a different color: red, supergroup A; blue, supergroup B; green, supergroup D; orange, supergroup F. The composite figure was created in https://BioRender.com, accessed on 12 February 2024.

Allelic profiles generated from these loci were compared against the *Wolbachia* MLST database (https://pubmlst.org/, accessed on 10 February 2024), confirming that all *Wolbachia* sequences obtained from the 20 *Cx. pipiens* samples corresponded to ST9, placing them within the *w*Pip clade of *Wolbachia* supergroup B (Table A2). The three hybrid individuals showed the same allelic profile. No novel alleles or mixed infections were detected in any of the samples. Phylogenetic analysis using concatenated MLST loci sequences (*coxA*, *gatB*, *ftsZ*, *fbpA*, and *hcpA*) and the *wsp*HVR supported these findings, reinforcing their classification within the *w*Pip lineage (Figure 2).

## 4. Discussion

### 4.1. The Prevalence of Wolbachia in Ae. aegypti

The absence of *Wolbachia* in all *Ae. aegypti* specimens analyzed in this study is consistent with most global surveys reporting a lack of natural infection in this species [35,36] and was confirmed using both *wsp* and 16S rRNA gene amplification. Although *Ae. aegypti* is not typically a natural host for *Wolbachia*, recent studies have detected low-density infections in wild populations under specific ecological conditions, suggesting that such infections, while rare, can occasionally occur [37]. For instance, low-prevalence *Wolbachia* infections have been reported in field-collected *Ae. aegypti* from the Philippines and India [37,38,39]. These findings indicate that natural infections in *Ae. aegypti* may emerge in localized contexts but remain uncommon. While *Wolbachia* has been successfully introduced into *Ae. aegypti* populations for vector control purposes, the feasibility of such strategies depends on the absence or very low prevalence of natural infections [40].

This study provides the first data on *Wolbachia* screening in *Ae. aegypti* populations from Funchal and shows relevance for potential *Wolbachia*-based interventions in Madeira, as the feasibility of such strategies depends on the need for artificial transinfection. If *Wolbachia* is to be introduced into Madeira’s *Ae. aegypti*, factors such as strain selection, host compatibility, and environmental stability must be considered [41,42].

Although PCR-based detection is highly sensitive, it is still possible that very low-density infections remain undetected [40]. Future studies could complement these findings using quantitative PCR (qPCR) or next-generation sequencing (NGS) to rule out low-level infections [43,44]. Given the confirmed presence of insecticide resistance in Madeira’s *Ae. aegypti* populations [23,24], alternative control strategies should continue to be explored.

### 4.2. High Wolbachia Prevalence in Cx. pipiens

Distinguishing between members of the *Cx. pipiens* complex can be challenging, as the two biotypes, *pipiens* and *molestus*, are morphologically identical, even in key structures such as the male genitalia. To overcome this limitation, we used molecular tools for accurate identification. The *ace-2* fragment analysis confirmed that none of the tested specimens belonged to *Cx. quinquefasciatus*, as all exhibited the expected 610 bp fragment characteristic of *Cx. pipiens* [26]. Subsequent analysis using the CQ11 microsatellite locus revealed that 37 individuals belonged to *Cx. pipiens* biotype *molestus*, while three mosquitoes showed both the 250 bp and 200 bp fragments, clear evidence of hybridization between the *molestus* and *pipiens* biotype [27]. To the best of our knowledge, this is the first report of *Cx. pipiens* hybrids on Madeira Island. Regarding the unexpected detection of hybrids despite no pure *Cx. pipiens* biotype *pipiens* specimens being identified, we believe this may be related to the nature of the sampling sites. Our mosquito sampling was indeed conducted in both urban and suburban areas within the municipality of Funchal, which predominantly favors the ecological niche of the *Cx. pipiens* biotype *molestus*. The predominance of the *molestus* biotype suggests that it is well adapted to the island’s urban environment, likely aided by traits such as its ability to reproduce without a blood meal (autogeny), to mate in confined spaces (stenogamy), and to remain active year-round without undergoing diapause [27]. These characteristics give this mosquito a clear ecological advantage in human-altered habitats.

The absence of pure *Cx. pipiens* biotype *pipiens* individuals during our sampling might indicate a very low abundance or limited distribution of this biotype in the areas surveyed. Further targeted studies are necessary to clarify this pattern.

This study represents the first molecular screening of *Wolbachia* in *Cx. pipiens* from Madeira Island, revealing a 100% infection rate across both 2023 and 2024 samples. The *Wolbachia* strain detected in all samples belongs to the wPip clade, within *Wolbachia* supergroup B, as confirmed by MLST typing. The detection of ST9 in all *Cx. pipiens* specimens from Funchal is consistent with previous findings from other parts of the world, particularly in Europe and North America. Similar studies have shown that *Cx. pipiens* s.l. populations commonly harbor wPip strains, often corresponding to ST9 or closely related sequence types [45,46,47]. These strains are known to belong to *Wolbachia* supergroup B and are associated with strong cytoplasmic incompatibility effects, which has direct implications for potential population suppression strategies such as the Incompatible Insect Technique (IIT).

Phylogenetic analysis of the concatenated MLST sequences (Figure 2) confirmed that all samples clustered within the wPip clade, a well-supported and genetically cohesive lineage within *Wolbachia* supergroup B. The topology of the phylogenetic tree and strong bootstrap support values validate the accuracy of sequence typing and reinforce the classification of the detected strains as ST9.

### 4.3. Implications for Vector Competence and Control

The detection of *Wolbachia* in Madeira’s *Cx. pipiens* has important implications for vector competence and vector control. The presence of hybrids is particularly noteworthy, as interbreeding between biotypes with different host preferences, the *pipiens* biotype tending to feed on birds and the *molestus* biotype tending to feed on mammals, including humans, may lead to mosquito populations with expanded host ranges and potentially greater capacity to transmit zoonotic pathogens like WNV [27].

Studies have shown that *Wolbachia* can influence pathogen transmission in *Cx. pipiens*, particularly for WNV and filarial nematodes [48,49]. However, the impact of *Wolbachia* on WNV transmission is complex, with some studies reporting reduced viral replication, while others suggest that it may enhance pathogen transmission depending on the specific *Wolbachia* strain–host interaction [50]. Understanding how *Wolbachia* affects WNV dynamics in Madeira’s *Cx. pipiens* is critical for assessing its role in arbovirus epidemiology.

Given the high prevalence of *Wolbachia* in Madeira’s *Cx. pipiens*, the Incompatible Insect Technique (IIT) remains a viable tool for population suppression [51]. Although all tested *Cx. pipiens* mosquitoes naturally harbored the wPip strain, IIT could still be effectively implemented by introducing males infected with a distinct and incompatible *Wolbachia* variant. Such variants can be obtained either through transinfection—artificial transfer of *Wolbachia* strains from other mosquito species—or by introducing naturally occurring incompatible wPip variants from geographically distinct populations via controlled backcrossing. This strategic introduction would induce cytoplasmic incompatibility and thereby significantly reduce mosquito fertility and ultimately lower population densities. Similar approaches have already proven successful in *Cx. quinquefasciatus*, where the release of incompatible *Wolbachia* strains significantly reduced mosquito populations and disrupted disease transmission cycles, including those of *Wuchereria bancrofti* [52].

Future research should focus on quantifying *Wolbachia* density using qPCR to evaluate how environmental factors influence bacterial load, which can directly affect pathogen inhibition and the degree of cytoplasmic incompatibility [53]. Additionally, vector competence studies are important to clarify the role of the naturally occurring *Wolbachia* ST9 infection *Cx. pipiens* population, particularly regarding WNV transmission dynamics. Although, based on the population assessed in the present study, *Wolbachia* prevalence in *Cx. pipiens* from Funchal currently stands at 100%, understanding potential variations in *Wolbachia* density is important, as it may significantly influence vector competence. Such studies will also help assess whether introducing novel *Wolbachia* strains, which might reduce WNV transmission more effectively compared to the natural strain, could be a viable strategy for vector management on the island.

## 5. Conclusions

This study represents the first screening of *Wolbachia* in *Ae. aegypti* and *Cx. pipiens* from Madeira Island. The fact that no evidence of *Wolbachia* infection was found in *Ae. aegypti* aligns with previous studies reporting the absence of natural *Wolbachia* infections in *Ae. aegypti* populations. The detection of *Wolbachia* in all tested *Cx. pipiens* biotype *molestus* and hybrid biotypes highlights their widespread prevalence in the local mosquito population, confirming the presence of the *w*Pip clade, supergroup B, ST9. Given *Wolbachia*’s potential to influence vector competence and population control, these findings contribute to the broader understanding of *Wolbachia* diversity in *Cx. pipiens* and its potential role in arbovirus transmission. Finally, our study emphasizes the need for continued surveillance and broader geographic sampling.

## Data Availability

The data supporting the results of this article are included in the paper. The nucleotide sequence data reported in this paper have been deposited in the NBCI GenBank database under accession numbers PV224315–PV224326.

## Abbreviations

The following abbreviations are used in this manuscript:

ace-2	Acetylcholinesterase-2
IIT	Incompatible Insect Technique
MBDs	Mosquito-borne diseases
MLST	Multilocus Sequence Typing
NGS	Next-generation sequencing
PCR	Polymerase chain reaction
qPCR	Quantitative PCR
ST	Sequence type
WNV	West Nile virus
*wsp*	*Wolbachia* surface protein gene

## Appendix A

**Table A1:** Primers used in this work for *Wolbachia* detection using PCR and for the amplification of the MLST complex and the *wsp*HVR.

Target	Primer Sequences (5′–3′)	Reference
*wsp*	81F-TGGTCCAATAAGTGATGAAGAAA 691R-AAAAATTAAACGCTACTCCA	[2]
*coxA*	wsp_F1: GTCCAATARSTGATGARGAAAC wsp_R1: CYGCACCAAYAGYRCTRTAAA	[3]
*gatB*	gatB_F1: GAKTTAAAYCGYGCAGGBGTT gatB_R1: TGGYAAYTCRGGYAAAGATGA	[3]
*ftsZ*	ftsZ_F1: ATYATGGARCATATAAARGATAG ftsZ_R1: TCRAGYAATGGATTRGATAT	[3]
*hcpA*	hcpA_F1: GAAATARCAGTTGCTGCAAA hcpA_R1: GAAAGTYRAGCAAGYTCTG	[3]
*fbpA*	fbpA_F1: GCTGCTCCRCTTGGYWTGAT fbpA_R1: CCRCCAGARAAAAYYACTATTC	[3]
*wsp*HVR	wsp_F1: GTCCAATARSTGATGARGAAAC wsp_R1: CYGCACCAAYAGYRCTRTAAA	[3]

**Table A2:** MLST allelic profiles, *wsp*HVR variants, and corresponding sequence types (STs) of *Wolbachia d*etected in *Cx. pipiens*.

Sex	Year	*gatB*	*coxA*	*hcpA*	*ftsZ*	*fbpA*	HVR1	HVR2	HVR3	HVR4	ST
Female	2023	4	3	3	22	4	10	8	10	8	9
	2024	4	3	3	22	4	10	8	10	8	9
Male	2023	4	3	3	22	4	10	8	10	8	9
	2024	4	3	3	22	4	10	8	10	8	9
